# Heading in Female Soccer: A Scoping Systematic Review

**DOI:** 10.3390/sports12120327

**Published:** 2024-11-29

**Authors:** Yinhao Shen, Shinting Chen, Qingguang Liu, Antonio Cicchella

**Affiliations:** International College of Football, Tongji University, No.1239, Siping Road, Yangpu, Shanghai 200092, China; 11089@tongji.edu.cn (Y.S.); 2132014@tongji.edu.cn (S.C.); qingguang@tongji.edu.cn (Q.L.)

**Keywords:** soccer, female, heading, kinematics, heading exposure, heading skills

## Abstract

Heading is a key skill in soccer, and it is few investigated in females. Research on heading focused mostly on males and on young players. Data on females’ soccer players are sparse and it is difficult to draw firm conclusions. Thus, little is known is known about heading in females. The most investigated aspects of heading are the relationship between heading and play state, training level and anthropometrics. The relationship between the frequency and intensity of headings and long-time outcomes in terms of vigilance, and neuro-cognitive status is also a topic of interest. Aim of this scoping review is to survey the available knowledge about heading in female football to identify possible weaknesses and issues for future research direction in the field. A structured literature search was performed in the main databases. Results show research on heading in female soccer is sparse and to draw firm conclusion on the investigated aspects (effect of play position, occurrence, cognitive impairment, influence of muscle strength, and player’s level) is difficult. It emerged mild intensity heading is not dangerous, helmet does not help, play state and player position influences the heading and that high rotational velocities are achieved. The survey identified new directions for research, that should focus on how to ameliorate heading training and skills and develop a more effective and safe heading technique.

## 1. Introduction

Women’s soccer is underrepresented in research, especially in biomechanics research [1]. Most of research investigating biomechanics has been performed in males. Despite the increase in female soccer players, there is a dearth of research in biomechanics in female soccer. Consequently most coaching advice provided to female athletes are solely based on data collected from male players [2], despite Males and females being different for range of motion and flexibility [3] body composition [4], and strength [5]. A large study on over 400 subjects [3], concluded females present greater flexibility (greater hip flexion) and balance than males while males showed higher isokinetic strength of lower limbs. Body fat is also different distributed in females and males’ bodies, having the female athletes more fat in the thigh [4]. Higher strength in man is more likely caused by larger muscle fibers than cross-sectional area [5]. The most serious injuries (as consequences) in soccer are those to the head. The general problem of uneven consideration of female injuries to the head was recently highlighted by a paper studying head injuries in crash tests in the automotive industries. This paper described the development of a dummy woman for automotive crash tests [6]. Before this, no such device existed for females, despite more than 50 years of research in crash injuries. For this purpose, was developed a 162 cm and 62 kg dummy, having the inertial characteristics of a mean western, middle aged, female body [6]. Heading in football is a cause of major concussive events, and women are more prone to concussive injuries than men [7]. Despite the high incidence of head injuries and concussive and sub-concussive outcomes, heading in female has been poorly studied from a biomechanical point of view. The microstructural and neurological changes that occur in the brain after continued exposure to heading in soccer players, seems to cause a more pronounced decline in the neurocognitive functions in females [8]. Another review study found biomechanical models of the neck were biased toward males [9]. The same study found female biomechanical research was biased in the height, weight, and BMI distributions [9]. Given the differences in inertial characteristics the head and neck between female and males, these factors influence the heading and its impacts [10].

Heading in soccer can be a frontal, lateral or by vertex impact. Lateral (rotational) heading for goal usually begins with a pre-load stretch [11,12].

The most important muscular motors acting in the heading are the cervical rotator muscles (scalene anterior, longus capitis and longus colli) which gives the highest contribution to the development of the rotational acceleration of the head [13]. In the specific task of lateral heading, before ball contact, there is also a pre-load rotation and a stretch phase of neck muscles [13]. Different indexes can be used to rate the chance of injury after an impact. One of the most accepted is the *HIC*, or head injury criterion.

The head injury criterion is commonly used and is described in the following equation [14]:

(1) $$\mathrm{HIC}=\frac{max}{t1,t2}{\left\{\left(t2-t1\right)\cdot [\frac{1}{t2-t1}\int_{t1}^{t2}a\left(t\right)dt]\right\}}^{2.5}$$

where *t*1 and *t*2 are the initial and final times (in seconds) chosen to maximize *HIC*, and acceleration *a* is measured in *gs* (standard gravity acceleration units). The time duration, *t*2 *− t*1, can be limited to a maximum value of 36 ms, where the mean impact value is 15 ms [15]. This means that the HIC includes the effects of head acceleration and the duration of the acceleration. Large accelerations can be tolerated for very short times. At a HIC of 1000, there is a 18% probability of a severe head injury, a 55% probability of a serious injury and a 90% probability of a moderate head injury to the average adult [16]. Concussions were found to occur at HIC = 250 in most athletes [17]. The mean HIC found in soccer heading in males was 133.3, thus far away from the critical value [18]. The aim of this scoping review is to analyze the existent literature about female head impact in female’s soccer players to provide values for head accelerations and velocities. A second aim is to explore the current directions of research in the field and to identify gaps to be filled in future research.

## 2. Methods

A comprehensive computerized search was performed: in the following databases: PubMed, Scopus, WoS, Sport Discus (Ebsco).

The following search strings were employed:

“heading in soccer” OR “heading in football” OR “heading in females soccer players” OR “soccer and heading” OR “gender and heading” OR “head impact in soccer” OR “female head impact in soccer” AND “team sport” OR football OR soccer OR futsal” OR “heading biomechanics” AND “female” AND “soccer”. The search items were selected for each database consulted. We consulted the MESH terms in PubMed, and searched for the keywords in article type, abstract and keywords in the Scopus database. In Wos we used the Descriptors, Gender, and Mesh headings to retrieve the papers. For Sport Discus we used the thesaurus keywords provided by the database. We found that all databases provided the same relevant keywords. Afterward, the search was performed by substituting soccer with “football” and different combinations of these terms were used.

The reference lists of the studies retrieved were manually searched to identify potentially eligible studies not captured by the electronic searches. Finally, an external expert was contacted to verify the final list of references included in this review and to check if there were any other relevant studies not detected through our search. As suggested by Cochrane’s guidelines, the inclusion and exclusion criteria were also provided to the external expert [19]. However, no information was provided about which databases to consult or search strategies to avoid creating a bias in the expert. This process (a double-check by an external expert) is recommended by PRISMA guidelines, to confirm the accuracy of the initial search. The databases searches included papers from the last 15 years up to August 2023. Only papers in scholarly journals indexed in SCIE. were considered. Paper older than 15 years were excluded from the search because the obsolete technologies used in the measurements cannot guarantee reliable data. The search was made in compliance with the PRISMA Checklist (see Supplementary file). Outcomes for which data were sough included head velocities and accelerations in different playing condition in experienced female soccer players. The review was registered in the PROSPERO database with nr. 613726.

### 2.1. Eligibility Criteria

The inclusion criteria of the present study, were as follows: (i) only head impact in females in soccer; (ii) study that included the mechanics of the heading in females, and outcomes (acute or long term) and (iii) published in the last 15 years. Furthermore, all the studies had to be peer-reviewed, written in English, and provide the full-text. Studies were excluded if they: (i) focused on males; (ii) were narrative reviews, brief reviews, scoping reviews or, methodological proposals; (iii) did not include relevant data in soccer headings; (iv) were not fully written in English; or (v) consisted of abstracts without accompanying full texts. Only studies on female subjects older than 14 years (to exclude pre-pubertal children) and with a sample size greater than 6 were considered. We choose the age range based on a previous study indicating this age is a discriminant in the onset of higher frequency of heading occurrence [20].

The screening of the title, abstract and reference list of each study to locate potentially relevant studies was independently performed by the two authors (AC and YS). Additionally, they reviewed the full version of the included papers in detail to identify articles that met the selection criteria.

### 2.2. Data Extraction

A data extraction sheet was prepared in Microsoft Excel (Microsoft Corporation, Readmon, WA, USA) in accordance with the Cochrane Consumers and Communication Review Group’s data extraction template [21,22]. The Excel sheet was used to assess inclusion requirements and subsequently tested for all selected studies. The process was independently conducted by the two authors. Full text articles excluded, with reasons, were recorded. All the records were stored in the sheet. Data were retrieved and checked by two authors. A specifically designed template for data extraction was developed. For each included heading, the following items were extracted: study citation details, purpose of the study, subject’s characteristics and anthropometrics, parameters measured and main outcomes. Means and standard deviations were calculated and reported for the kinematics parameters considered.

### 2.3. Data Items

The following information was extracted from the included heading studies: number of subjects included (n), age group (>14 years old), sex (women), competitive level (if available), and type of original articles included (experimental, observational analytic or both); identification of the effects if any (acute or adaptations), dimension of analysis (game situation, outcomes, kinematics) and main findings.

The methodological quality of the included studies was evaluated using the AXIS Tool for the Critical Appraisal of Cross-sectional Studies [23]. This tool contains 20 questions, used to assess the study design quality and risk of biases. Each question can be answered as ‘Yes’, ‘No’ or ‘Unsure/comment’. This assessment was performed independently by two reviewers. Where consensus could not be reached through discussion, a third reviewer was consulted.

## 3. Results

### Study Identification and Selection

**Figure 1:**
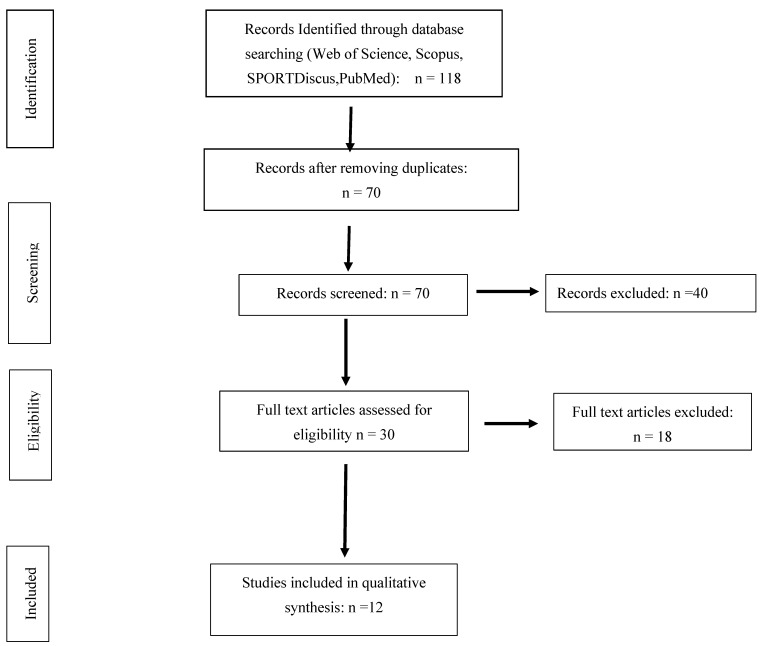
PRISMA flow chart.

The search produced a total of 118 papers. The references were then exported to reference manager software (EndNote^TM^ X9, Clarivate Analytics, Philadelphia, PA, USA). Duplicates (48 references) were subsequently removed either manually or automatically. The remaining 70 articles were screened for their relevance by reading the titles and the abstracts, and this process resulted in the removal of a further 40 studies. Following the screening procedure, 30 articles were selected for in depth reading and analysis. After reading the full texts, a further 10 studies were excluded due to not being heading studies (n = 3), not being written in English (n = 5), having fewer than six subjects (n1) and being performed with subjects younger than 14 years old (n = 1). Of the 12 papers included in this review, all were biomechanical and considered the outcomes. The AXIS analysis is reported in Figure 1.

**Figure 2:**
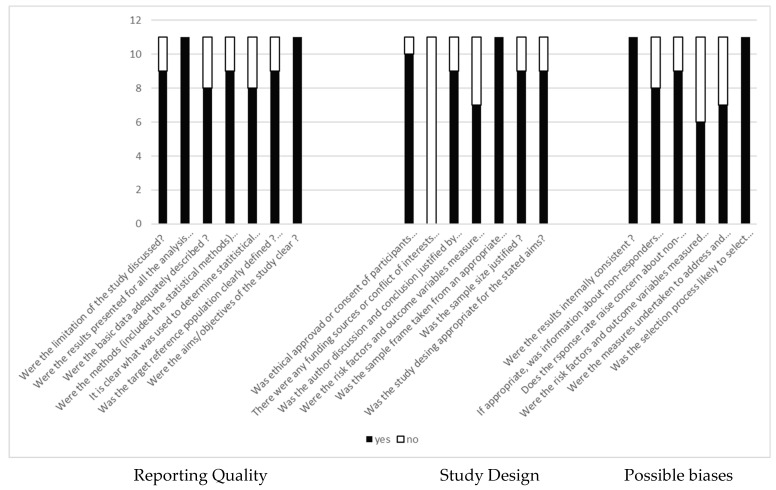
AXIS analysis of the studies. Study characteristics and qualitative synthesis.

In total, 12 studies that met the inclusion criteria were found. We classified the AXIS quality scores according to the number of “Yes” responses for the 20 items for each study. Studies achieving 80% “yes” responses indicated high quality, 60–80% indicated moderate quality, and less than 60% indicated low quality. Thus, studies rated as high quality were 4/12 (36.6%), moderate quality were 5/12 (36.6%), and low quality were 3/12 (27.7%). While the items relating specifically to reporting quality scored quite highly, the detail relating to study design and possible biases are lower and more variable (Figure 2).

The type of study (O = observational, E = experimental) and subjects characteristics are reported in Table 1.

Table 2 shows, the instrument used (MMS = Mouth Mounted Sensors; HMS = Head Mounted Sensor) the heading frequency, the main outcomes, and the variables measured. FA: forward acceleration; LA: left acceleration: RA: rotational acceleration; RV: rotational velocity.

PLA: peak lateral acceleration; PRA: peak rotational acceleration; PRV: peak rotational velocity.

Among the retrieved studies, 11 were published after 2017, showing an increasing interest in women’s safety in football in recent times. The summary of the main evidence found in each review can be found in Table 1. Most studies tested single groups or parallel, non-randomized groups. Seven studies employed a mouthpiece sensor, and five a head mounted sensor. Three studies were observational, and nine were experimental.

Of the included articles, we compared the magnitude of impact between practice and match setting, one studied the differences between male and females, two aimed at characterize the heading in relation to different play scenarios, one studied the differences in impact between young and experienced players, three studied the effect of measured parameters on injury and neurocognitive functions and the relationship between impact characteristics and the likelihood of developing a neurocognitive deficit, two correlated neck muscle and strength with the magnitude of the impact. Two analyzed which kick produced a higher impact in heading and one studied the dumping effect of headgear.

The mean sample size was 16.2 ± 8.7 subjects (range 7–34). Age, weight and height of the studied subjects were respectively: 18.8 ± 1.7 years, 62.3 ± 2.9 kg and 167.1 ± 3.27 cm (BMI 23.3 ± 2.3). 1 study didn’t report weight and height. Seven studies used NCAA div 1 players, two used high school players, one used players with >5 years of experience, one used varsity and one used under 15 teams.

The measured impact parameters were: Peak Linear Acceleration (PLA; m/s^2^); Mean Rotational Acceleration (RA; m/s^2^) and Mean Rotational Velocity (RV; m/s). One study reported frontal acceleration and lateral acceleration (FA; and LA m/s^2^). The mean values for PLA, RV and RA were 17.61 ± 6.27 m/s^2^; 25.84.6 ± 16.28.41 m/s and 8.27 ± 1.68 m/s^2^. Six studies reported the frequency of heading using four different metrics (per training session, per athletes’ exposure time, in training and in game). Exposure to heading ranges from 2 to 10 per match/training unit. The measurement devices used were essentially of two types: head and mouth mounted sensors. The main outcomes were that goal heading produced higher rotational acceleration in comparison to other games scenarios [24] and game situations influence heading kinematics [28]. Other results were that no difference was found between males and females in heading kinematics [25], the magnitude of heading was found to be higher during practices than game [26], and experienced players produced higher acceleration and velocities [27]. One study found that medium intensity soccer heading didn’t produce significant impairment of neurocognitive function in the short and long term [29,30]. The authors of [31] found that wearing a soft cells helmets increase PLA. Other studies found that head mass and neck girth negatively correlate with PLA and RV [32], and increasing the strength of the neck muscles mitigated PLA [35]. It was also found that the response to long kick produce higher magnitude kinematic response [33,34].

## 4. Discussion

Although heading is a relatively rare event in a soccer game compared with other skills, the health consequences of heading are of major concern and are the most investigated topic in the literature. Thus, the underlying motivations of several of the examined studies were the short and long-term outcomes of heading exposure. We noticed a comparison of heading frequencies between the studies was difficult, because of the use of different indexes. Rotational acceleration was the most studied parameter of heading. Differently from other sports, soccer is the only situation where maximal rotational acceleration is actively pursued in attempt to score a goal. In other sports, rotational acceleration is passive and caused by an external perturbation which is either expected as in boxing [36] or unexpected, as in a car crash [6]. The heading occurrence we found in females is similar to another study that showed an exposure of 3.6 to 8.6 headings per match [37]. However, within the context of epidemiological studies, it is unclear how to formulate an appropriate exposure metric. It has been proposed that estimates of the cumulative number of heading impacts over a playing career should be used as the main exposure metric in epidemiological studies of professional players [10]. The present state of knowledge does not support the fact that heading in female soccer players is deleterious and this result align with other review studies that focused on long term outcomes [38]. However, due to the relevance of the topic for health and safety, further studies focusing on female players are necessary: the existing studies on females are too few to draw a firm conclusion. Also, it concerns the question of how much risk is personally and socially acceptable, given that heading can also cause fatalities or permanent disabilities and cognitive problems at older ages. It emerged from the literature that mastering the heading skill was found in more experienced players. Although little is known about neck muscle in females, it was shown that when normalized for body mass, the neck muscle mass of females does not differ from males [39]. Protocols for strengthening the neck muscle has been evaluated in different sports [40], but we only found two studies on this topic, that showed the need to increase the muscle strength of the neck in females. On-field tactics during a match influence heading occurrence, thus suggesting adopting different tactical arrangement in the presence of a strong heading player. A player’s skills are also reflected in heading kinematics.

The available data about kinematics of the soccer heading in females are very scarce and dealt with the heterogenous aspects of heading. A limitation of our review was that we could not access non-English literature, and we did not review the coaching literature, which may contain some suggestion for further studies. For each direction of research, we found a maximum of two papers. The investigated aspects are sparse and there is no sound body of knowledge on the different aspects of heading in female football players. However, some evidence suggests that mild impact heading is not dangerous, head gears would not help in preventing injuries, and the match situation influences heading. It remains a problem that heading can cause serious outcomes in females. Further research is necessary to constitute a clear body of knowledge on female soccer players heading. We identified the following research directions for female soccer headings: (1) research connected to safety, and especially long-term observational studies of heading outcomes and the effect of the use of protective devices; (2) research connected to performance, e.g., intervention studies aimed at modifying of the heading biomechanics by increasing the strength of the neck muscles or ameliorating the pose before heading in response to different games situation (heading techniques interventions).

## 5. Conclusions

Further research is needed on female soccer players, to improve the existing knowledge about heading safety and improve performance. Furthermore, the gap between males and females’ players in this research field must be filled. Future research on biomechanics of headings on females should consider a more in-depth analysis of the effects on soft tissue, using simulation methods to build reliable models of a female’s head and implement the measured accelerations. This approach could reduce the need for direct measurement on the field and allow for broader and more complex experimental conditions in vivo.

## Figures and Tables

**Table 1:** Type of Study and subjects characteristics.

Author	Aim of the Study	Study Type	Mean Age (Years)	Weight (kg)	Height (cm)	Gender	Subject Number	Qualification Level
Filben et al. [24]	Categorize head impacts	O	19	62.3	170	F	16	NCAA div 1
Reynolds et al. [25]	Quantify head impactt	O	20.43	n.r.	n.r.	F	7	NCAA div 1
Lynall et al. [26]	Frequency and magnitude	O	19.1	63.7	168	F	22	NCAA
	of impact							
Filben et al. [27]	Difference between younger	E	15.27	61.8	n.r.	F	6	U15
	and experienced	E	20.19	63.08	n.r.	F	13	NCAA div 1
Caccese et al. [28]	Differences in PLA, RV		19.6	60.3	167.8	F	16	NCAA div 1
	in different heading scenarios							
Gutierrez et al. [29]	Effect of heading	E	15.9	59	165	F	17	HIGH SCHOOL
	on neurocognitive function							
Nowak et al. [30]	Assess the reliability	E	19.7	62	168.3	F	23	COLLEGE
	of concussion diagnosis							>5 yrs
Tierney et al. [31]	Headgear effect of PLA	E	19.5	63.2	164	F	29	>5 yrs
Muller et al. [32]	Asses neck and trunk strengthening	E	16.5	56	168	F	7	HIGH SCHOOL
Kenny et al. [33]	Which kick produced more impact	E	19.9	65.5	169.3	F	13	VARSITY
Mihalik et al. [34]	Characterize the impact	E	19.8	64	168.2	F	34	NCAA div 1
Bretzin et al. [35]	Compare PLA and RV	E	20.25	66.9	158.7	F	8	NCAA div 1
	with muscle strength							

**Table 2:** Instruments used, heading frequencies, outcomes, type of measure and results.

Author	Instrument	Heading	Outcomes	Measures	PLA	RA
		Frequency				
		(if reported)		PLA, PRA, PRV	(G)	(rad/s^2^)
Filben et al. [24]	MMS		Goal kicks	corner	22.9	2189.3
			shows higher PRA	goal	24.3	2658.9
				free	18	1843.3
				live	18.8	1769.7
Reynolds et al. [25]	MMS	5.7/practice	No difference	PLA, PRA	19.1	3687
			compared with men in severity			
Lynall et al. [26]	MMS	7/90 min game	More impacts	PLA, PRA	12.51	2093
			during practice			
Filben et al. [27]	MMS		Experienced players	PLA, PRA, RV	11.7	9450
			shows higher		17.3	1560
			PLA, PRA and RV			
Caccese et al. [28]	HMS		Different field scenarios	PLA, RA	28.2	7100
			Influence PLA and RA			
Gutierrez et al. [29]	HMS		descriptive study	FA	5.83	
				LA	6.96	
				RA	6.27	
Nowak et al. [30]	HMS		Head impact predicting	PLA	16.1	
			concussion outcomes			
Tierney et al. [31]	MMS		Head gear increase PLA	PLA	21.52	
Muller et al. [32]	HMS	1.9/90 min	Strength of neck	PLA	10.9	
			muscle mitigate PLA			
Kenny et al. [33]	MMS	1.83/Athlete	Long kick	PLA, RA	22.2	2296
		exposure time	=higher impact			
Mihalik et al. [34]	MMS	10/Session	Mild intensity impacts	PLA, RA	19	3457
			are not dangerous			
Bretzin et al. [35]	HMS		Head mass and neck girth	PLA, RV	24	1416
			negatively correlate			
			with PLA and RV			

## Supplementary Materials

The following supporting information can be downloaded at: https://www.mdpi.com/article/10.3390/sports12120327/s1, Supplementary File: Reporting checklist for systematic review.
